# Supplementary material for: Development of Sustainable Chemistry in Madagascar: Example of the Valuation of CNSL and the Use of Chromones as an Attractant for Mosquitoes
Source: Molecules. 2021 Dec 16;26(24):7625. doi: 10.3390/molecules26247625 (PMC8704927; doi:10.3390/molecules26247625)
Supplement: Supplementary file 1 [file molecules-26-07625-s001.zip › molecules-1473848-supplementary.pdf]

Supporting information: Sustainable Chemistry: An Opportunity for the Development of Madagascar

1- Cashew nut shell liquid valorization as surfactant

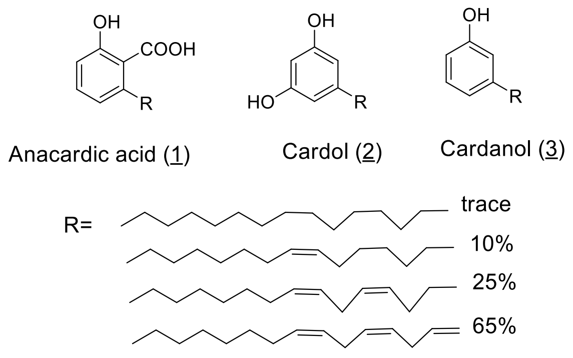

Figure S 1. Composition of CNSL

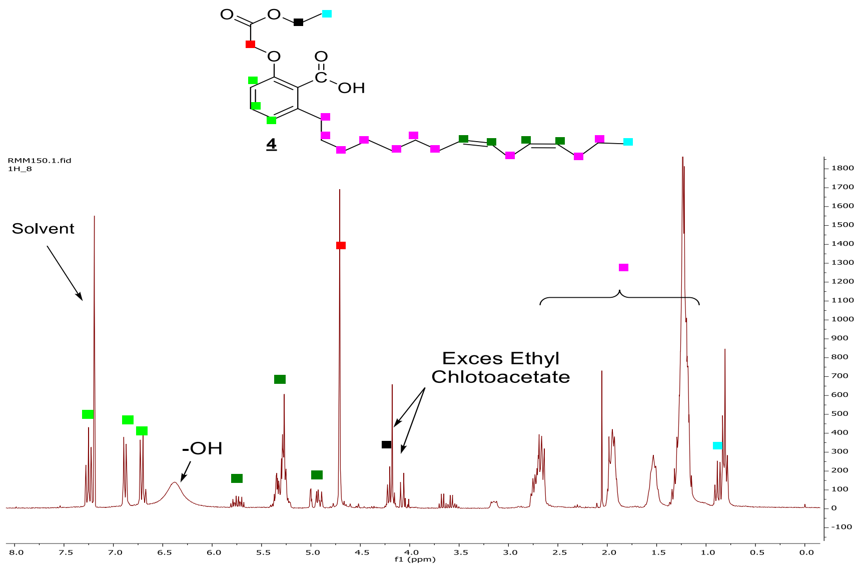

Figure S 2. NMR 1H Spectrum of Ester oxyacetic of anacardic acid (4)

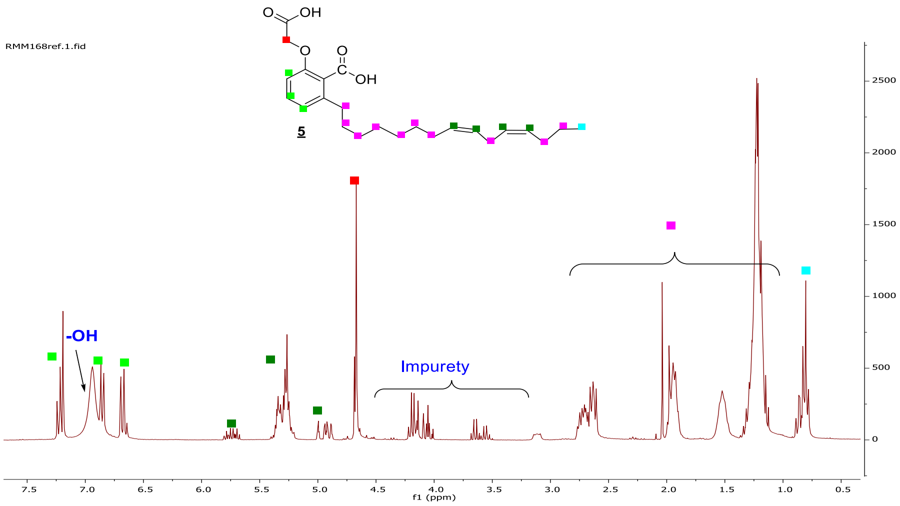

Figure S 3. NMR [1]H Spectrum of oxyacetic acid of Anacardic acid (5)

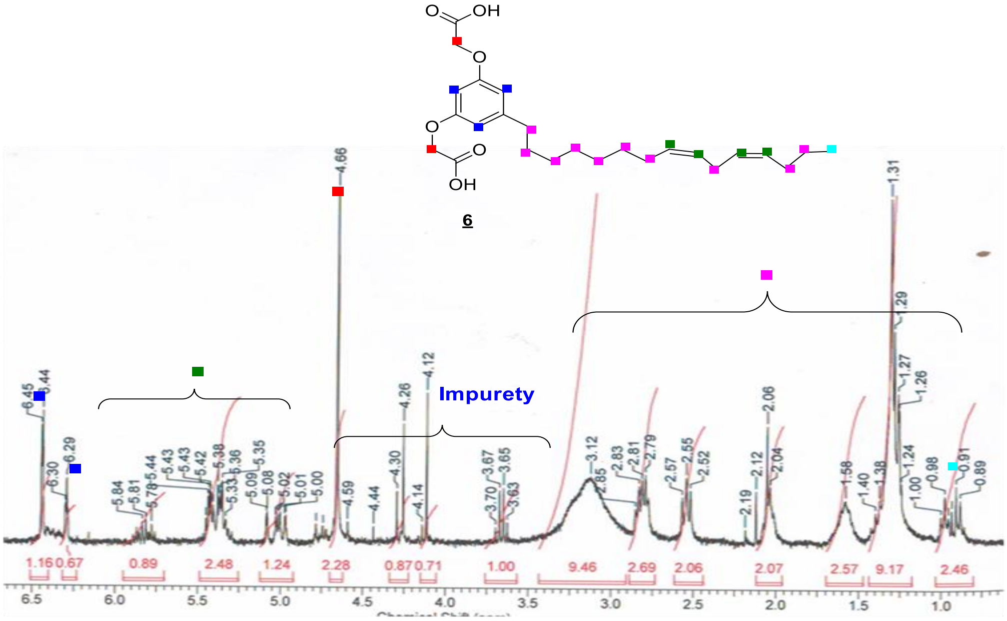

Figure S 4. NMR [1]H Spectrum of oxyacetic acid of cardol (6)

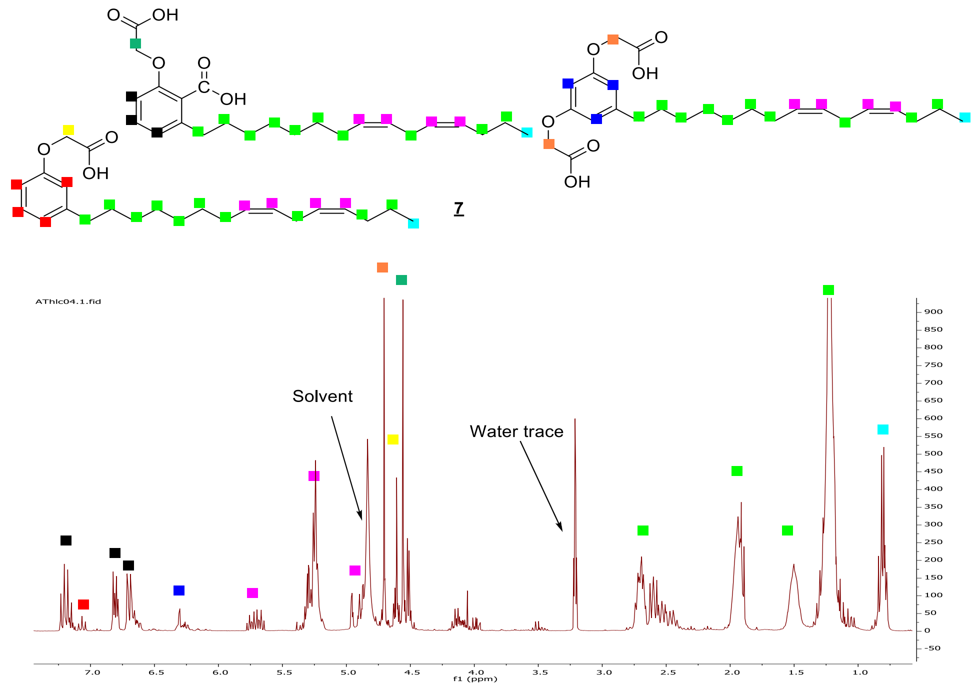

Figure S 5 . NMR ^{1}H Spectrum of oxyacetic acid of CNSL (7)

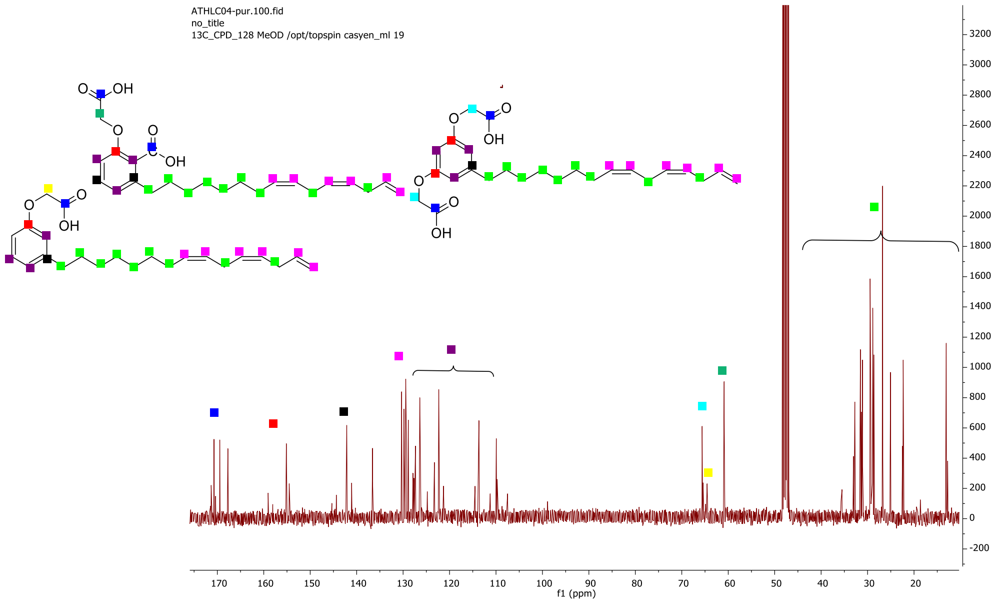

Figure S 6. NMR ^{13}C Spectrum of oxyacetic acid of CNSL (7)

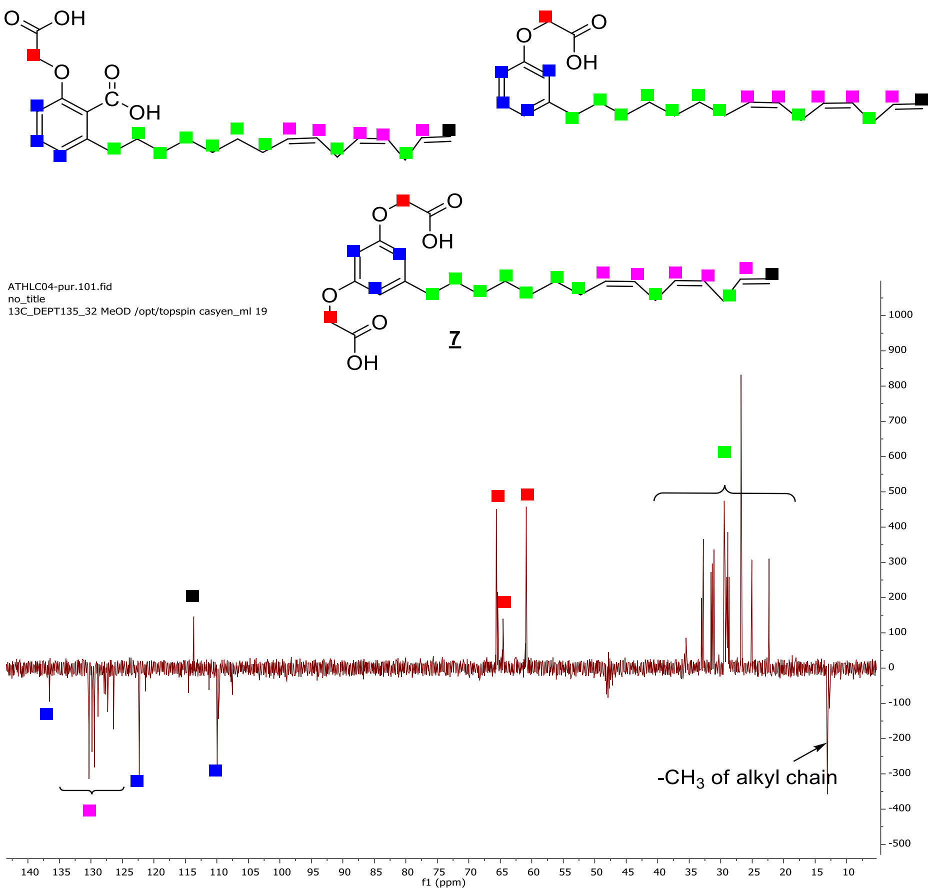

Figure S 7. NMR ^{13}C DEPT 135 Spectrum of oxyacetic acid of CNSL (7)

Table S1 : Data of the fluorescence emission spectrum of pyrene.

log (C)	I1/I3
-1,52287875	1,50351794
-1,39794001	1,48414432
-1,30103	1,43341818
-1	1,43402342
-0,30103	1,18944555
0	0,91672214
0,69897	0,75198674
1	0,6601263

Table S2 : Data of Foaming property of of Sodium oxyacetate of CNSL (8) and reference surfactant

Time (min)	Foam volume	Foam volume (mL)	Foam volume (mL) of Sodium

	(mL) of SDS	of LABSA	oxyacetate of CNSL (8)
0	220	210	200
0,5	190	200	180
1	170	180	160
1,5	150	150	120
2	120	120	80
2,5	100	80	60
3	80	60	40
3,5	60	40	40
4	40	30	40
4,5	20	20	40
5	10	20	30
5,5	10	10	30
6	10	10	30
6,5	5	10	20
7	5	10	20

2- Chemical ecology as alternative to insecticides

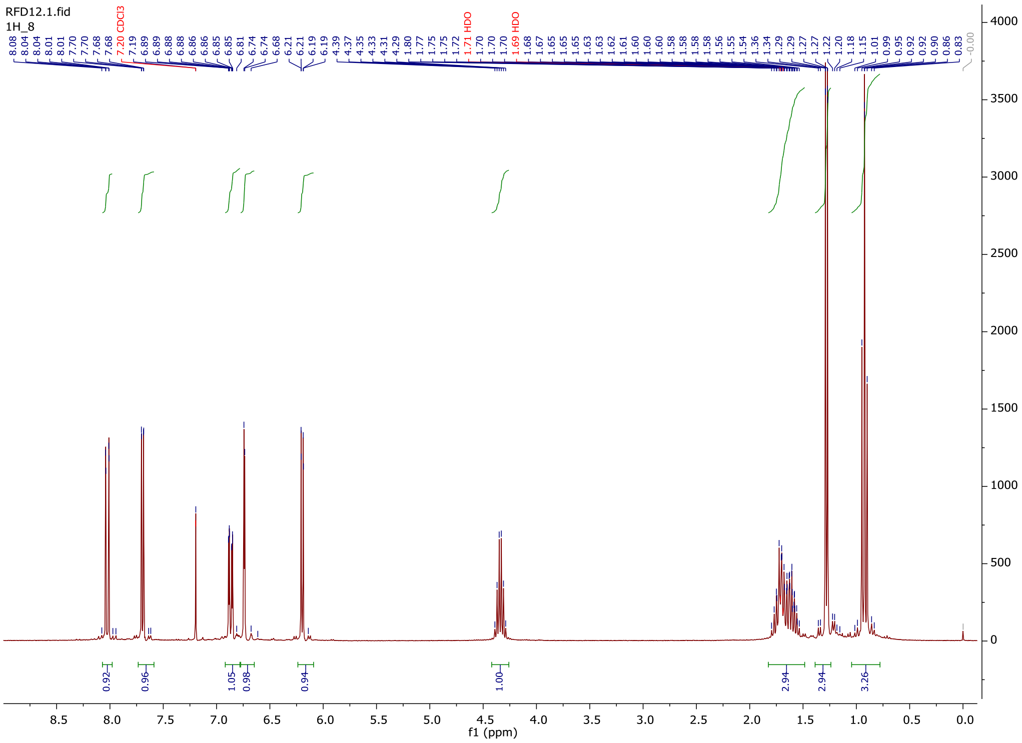

Figure S 8. NMR ^{1}H Spectrum of Racemic 7-*sec*-butoxychromone (10)

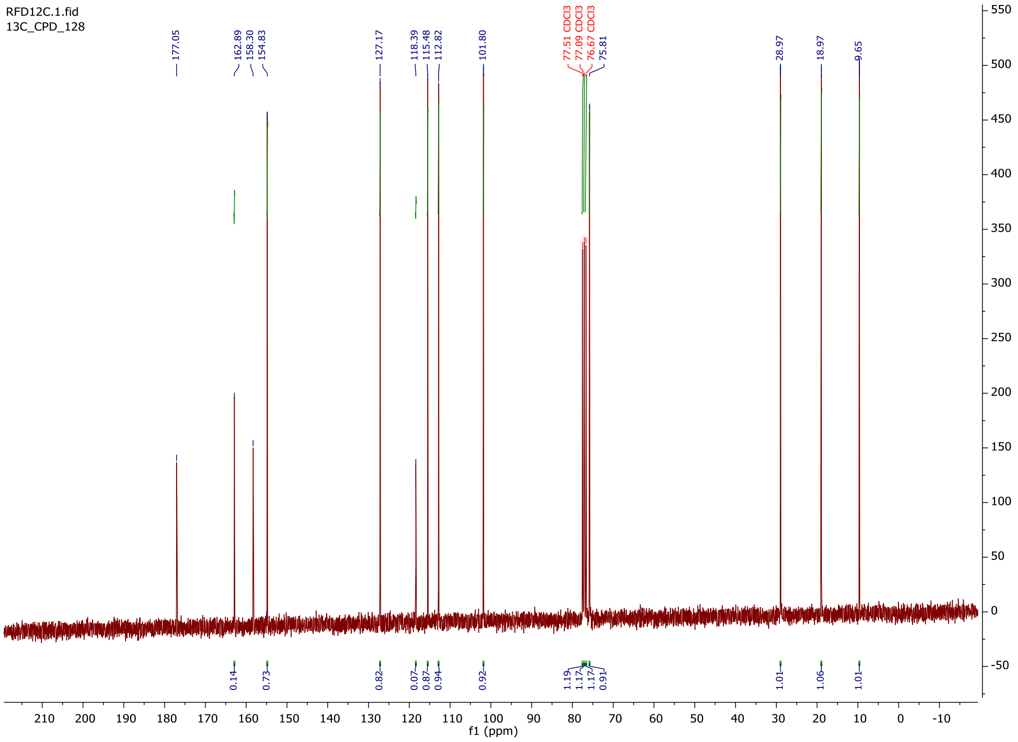

Figure S 9. NMR ^{13}C Spectrum of Racemic 7-*sec*-butoxychromone (10)

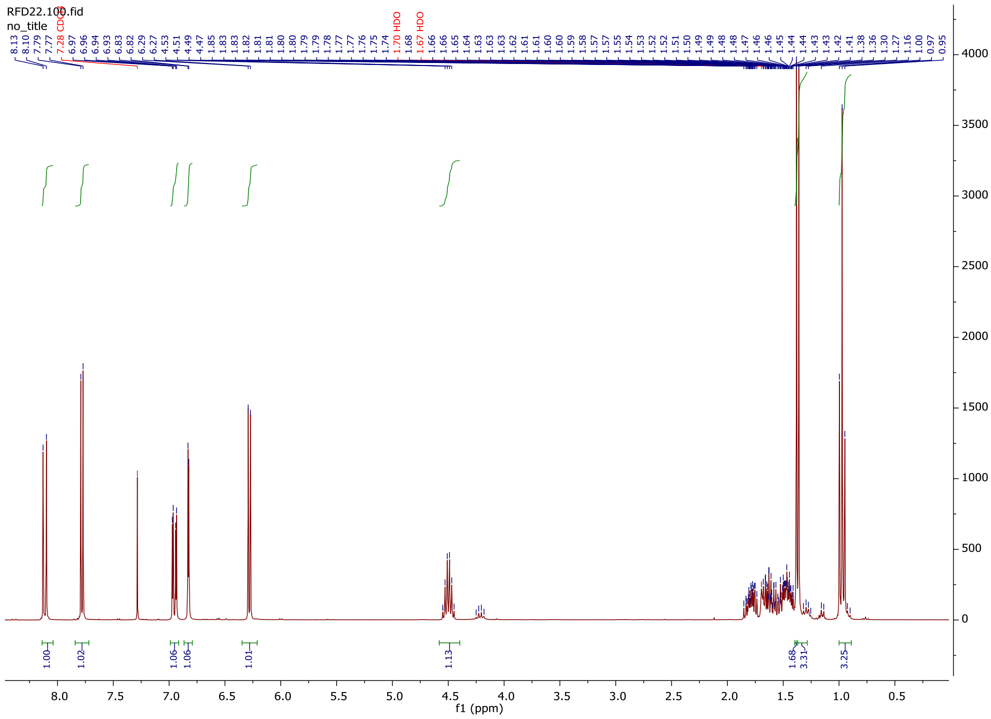

Figure S 10. NMR ^{1}H Spectrum of Racemic 7-*sec*-pentoxychromone (11)

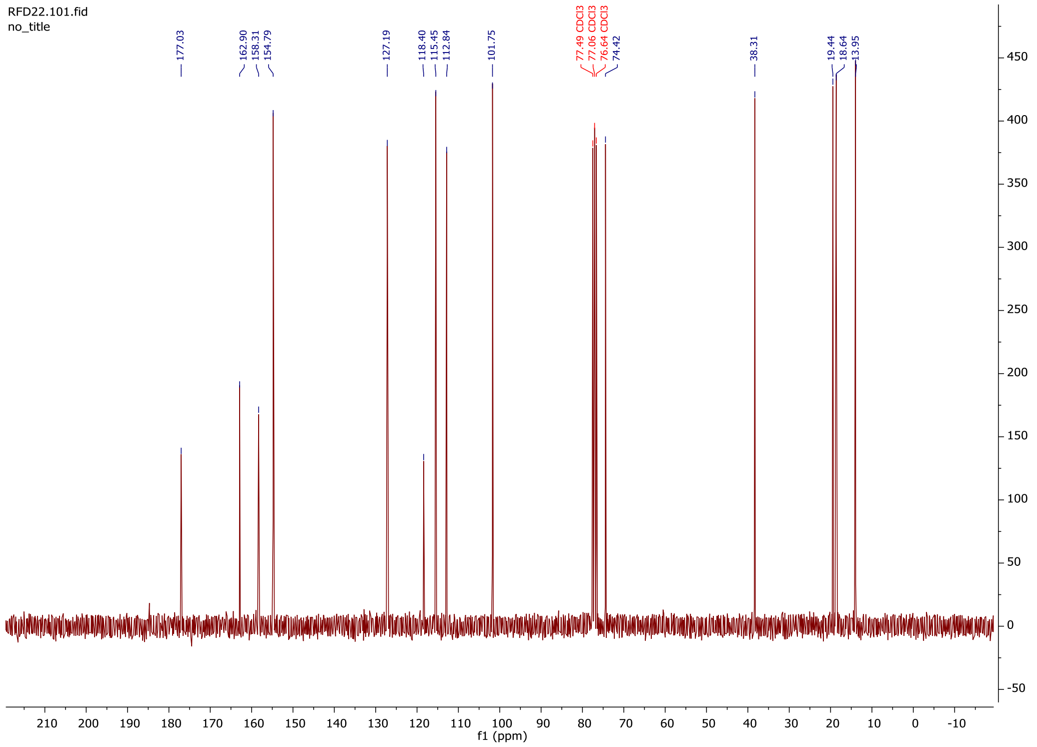

Figure S 11. NMR ^{13}C Spectrum of Racemic 7-*sec*-pentoxychromone (11)

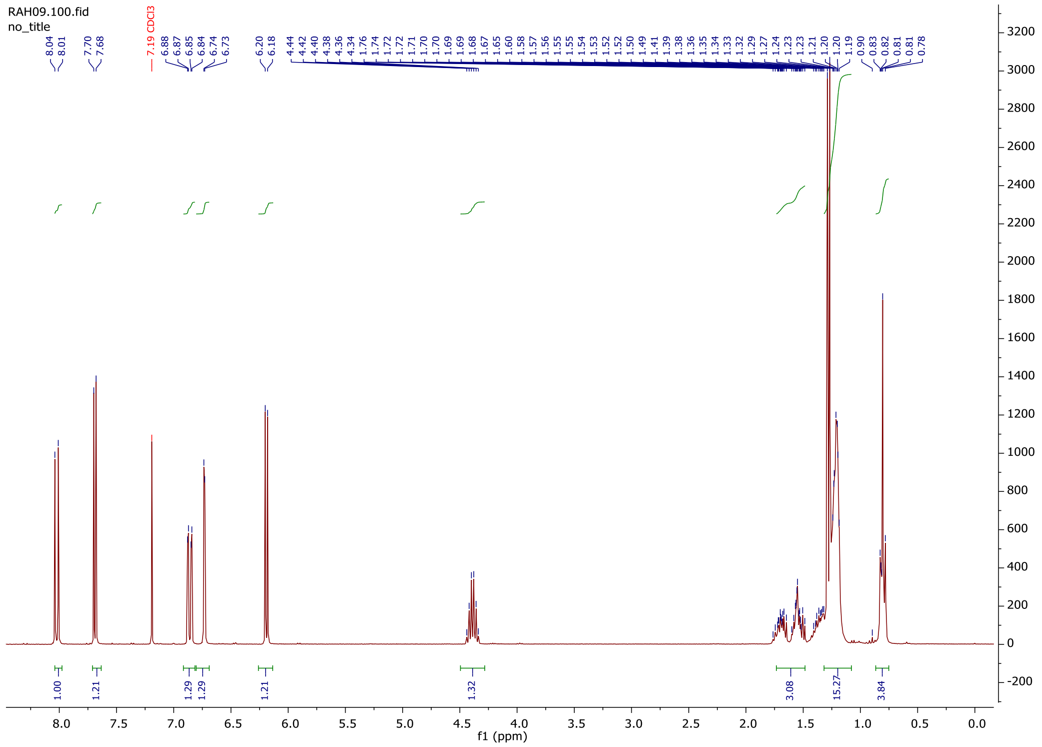

Figure S 12. NMR ^{1}H Spectrum of Racemic 7-*sec*-nonyloxychromone (12)

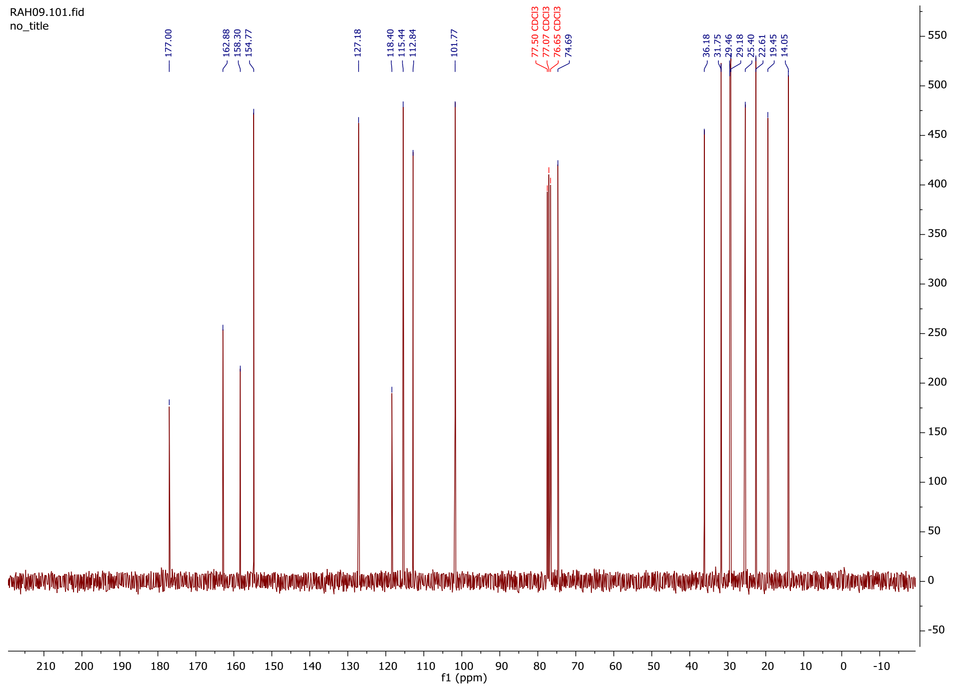

Figure S 13. NMR ^{13}C Spectrum of Racemic 7-*sec*-nonyloxychromone (12)

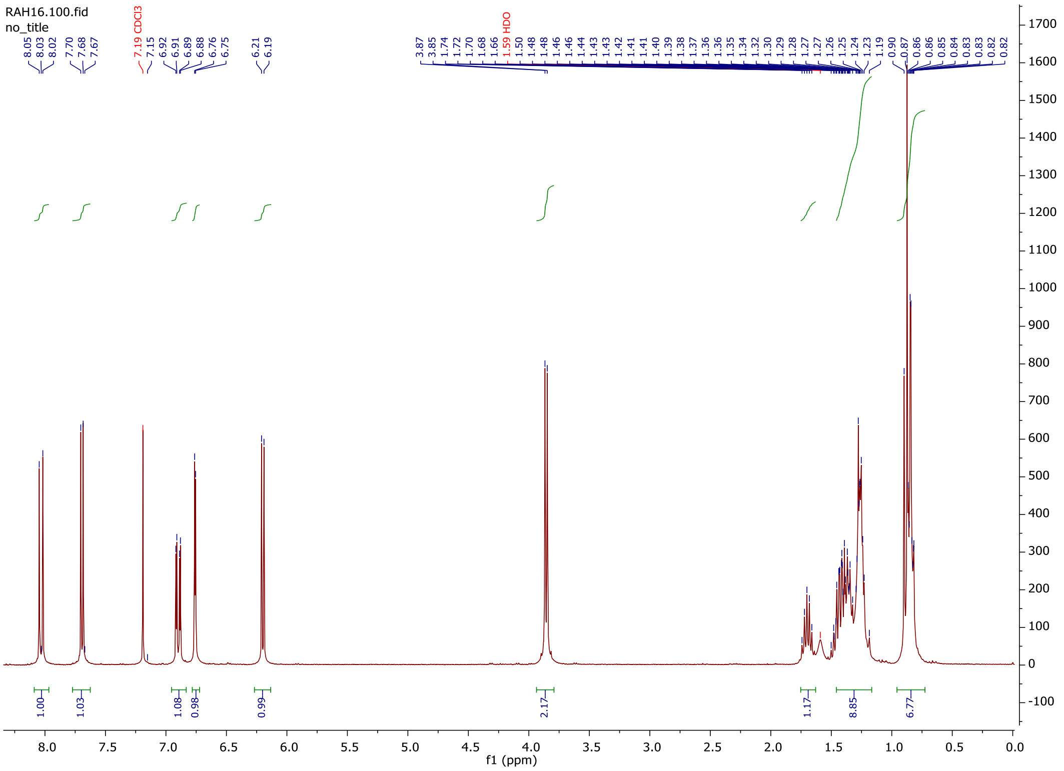

Figure S 14. NMR ^{1}H Spectrum of Racemic 7-(2′-ethyl)hexyloxychromone (13)

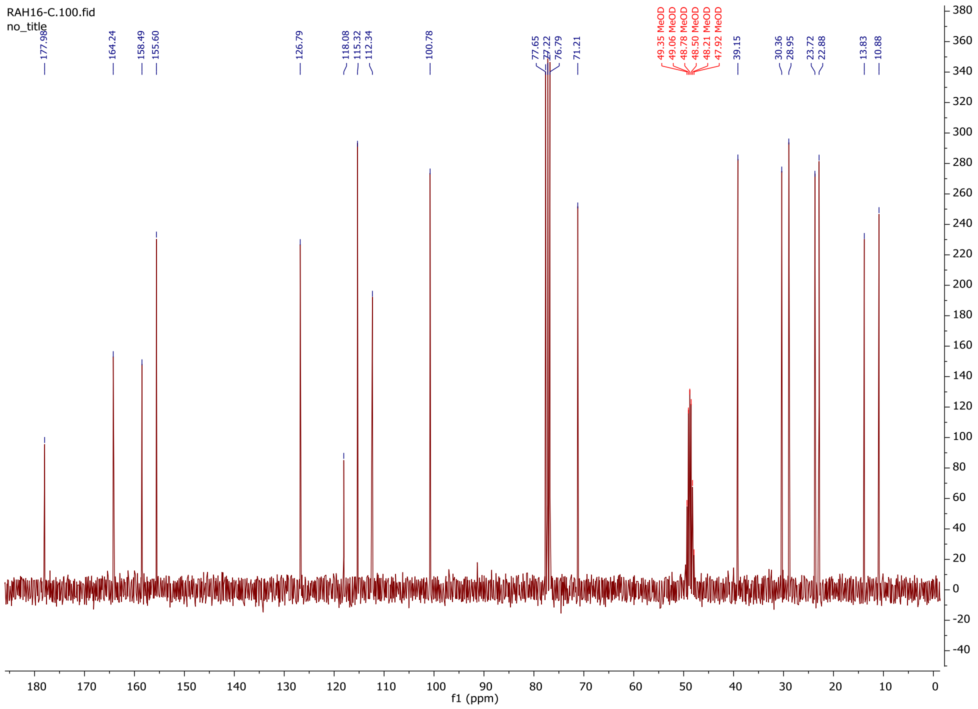

Figure S 15. NMR ¹³C Spectrum of Racemic 7-(2′-ethyl)hexyloxychromone (<u>13</u>)
